# Supplementary material for: MICRON learns outcome-associated representations of spatial immune microenvironments
Source: bioRxiv. 2026 Apr 16:2026.04.14.718488. Preprint. [Version 1] doi: 10.64898/2026.04.14.718488 (PMC13104970; doi:10.64898/2026.04.14.718488)
Supplement: Supplement 1 [file NIHPP2026.04.14.718488v1-supplement-1.pdf]

Supplementary Material for MICRON learns outcome-associated representations of spatial immune microenvironments

1. MICRON AND RELATED FEATURIZATION APPROACHES FOR IMC ANALYSIS

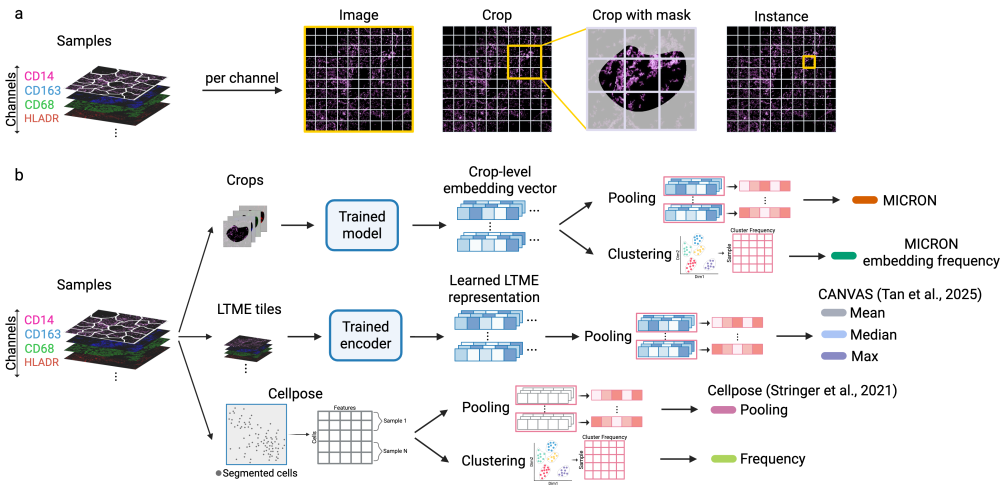

Fig. S1. Overview of MICRON and comparison to Cellpose-based and CANVAS-based featurization approaches. (**a**) Illustration of how image regions are defined in MICRON. Multiplexed imaging samples consist of multiple channels. Each sample is cropped into several square regions, where each crop contains multiple instances. A bounding box binary mask is generated by SLIC and applied to define the region of interest within each crop. (**b**) Comparison of MICRON with tile-based and segmentation-based approaches. In MICRON, each crop-level embedding vector is generated by a trained model. These embeddings are aggregated either by pooling to produce a sample-level representation or by clustering to compute embedding frequency features. In the CANVAS framework, local microenvironment (LTME) tiles are extracted and encoded using a trained encoder. Sample-level representations are obtained via global pooling operations (mean, median, or max) across tile embeddings. To define features via CellPose segmentations, the expression of each protein can either be pooled over all segmented cells or cells can be clustered to define frequency features.

2. ADDITIONAL BIOLOGICAL INSIGHTS GLEANED ACROSS DATASETS

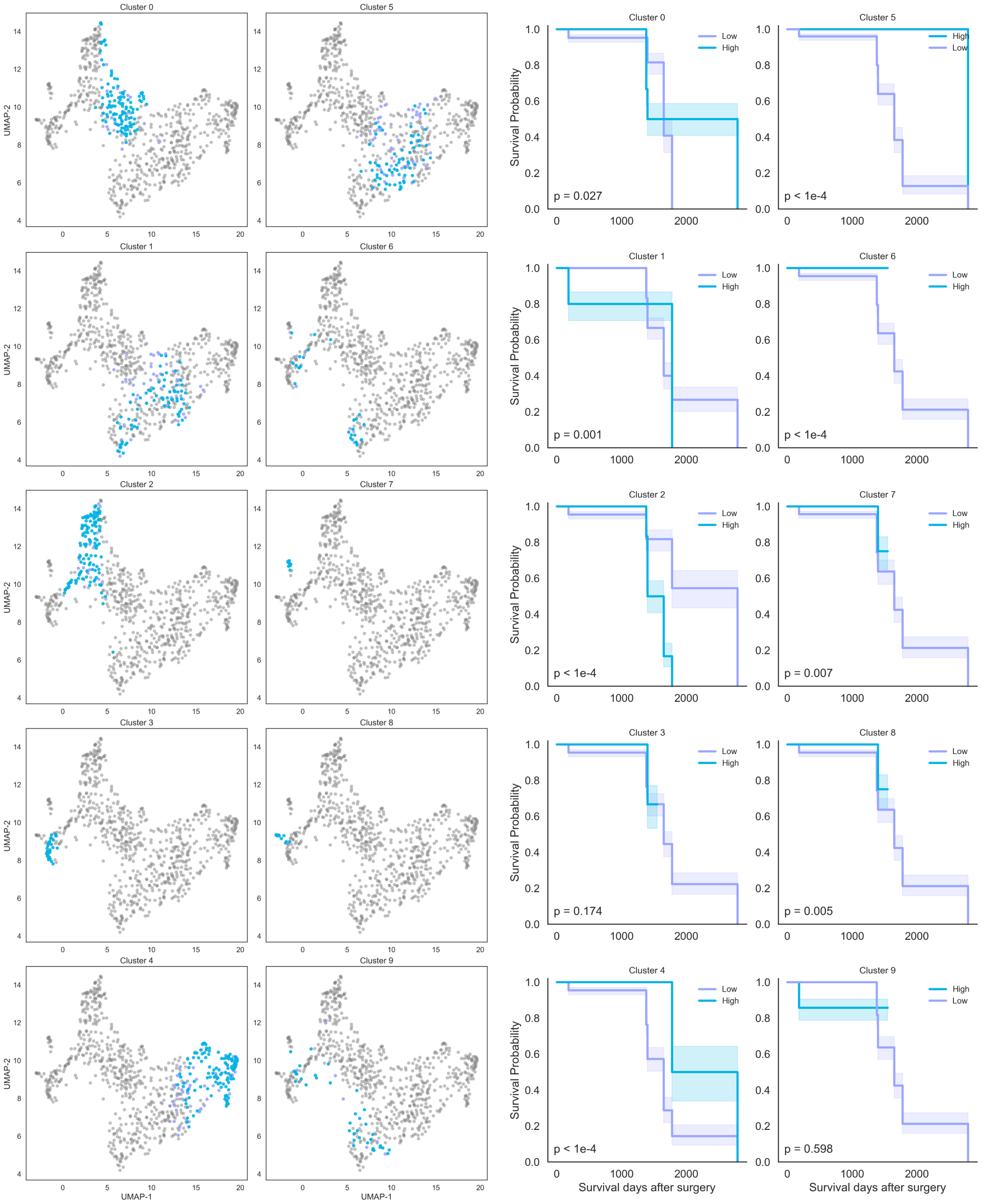

Fig. S2. Identifying survival-associated microenvironments. To evaluate the prognostic relevance of the learned superpixel representations under `MICRON`, we clustered superpixels across samples using k-means ($K = 10$) and computed the frequency of each cluster per patient. For a given cluster, patients were stratified into high- and low-frequency groups based on the 75th percentile frequency of that particular cluster. The UMAP visualizations provide two-dimensional projections of superpixels identified across images. Blue points denote cells belonging to the indicated cluster. Kaplan–Meier survival analysis was used to quantify the differences in survival between patients with high and low frequencies of a given cluster. Several clusters showed significant survival differences between high and low frequency groups. Cluster 4 showed the most significant association with survival ($p < 10^{-4}$).

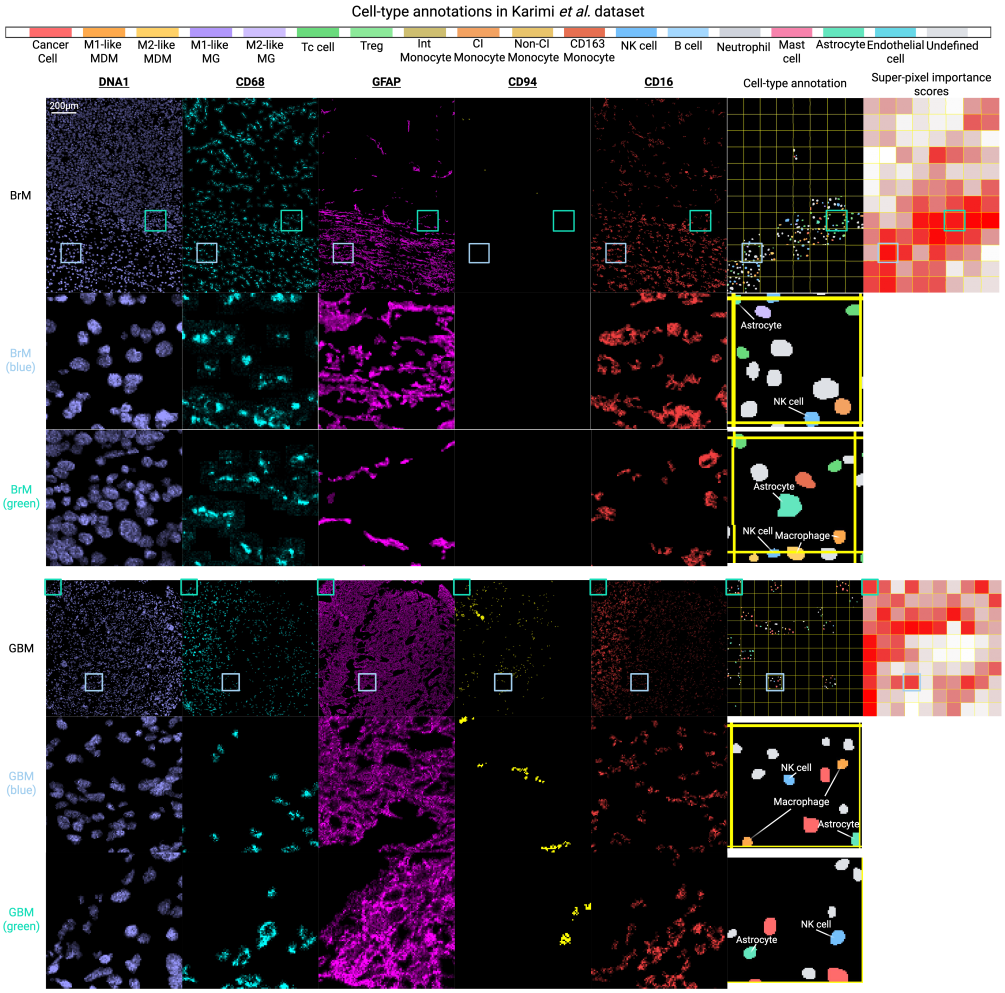

Fig. S3. Visualization of high-importance superpixel regions and associated cell-type markers in the Karimi brain tumors dataset. Representative superpixel regions with high importance scores identified by `MICRON` are shown for brain metastasis (BrM) and glioblastoma (GBM) samples (heatmaps on right). Images show expression for individual channels, including DNA1, CD68, GFAP, CD94, and CD16. DNA1 used to identify nuclei and determine cellular localization. Canonical markers were used to define key cell types, including $CD68^+$ macrophages, $GFAP^+$ astrocytes, and $CD94^+CD16^+$ NK cells. Blue and green squares denote two different superpixels selected and deemed to be outcome-associated. Corresponding cell-type annotations illustrate the spatial distribution of annotated cell populations. Zoomed-in views reveal spatial proximity among astrocytes, macrophages, and NK cells within the selected regions. Although CD94 expression was relatively low in BrM samples, $CD94^+$ signals were occasionally present but visually subtle in the corresponding images.

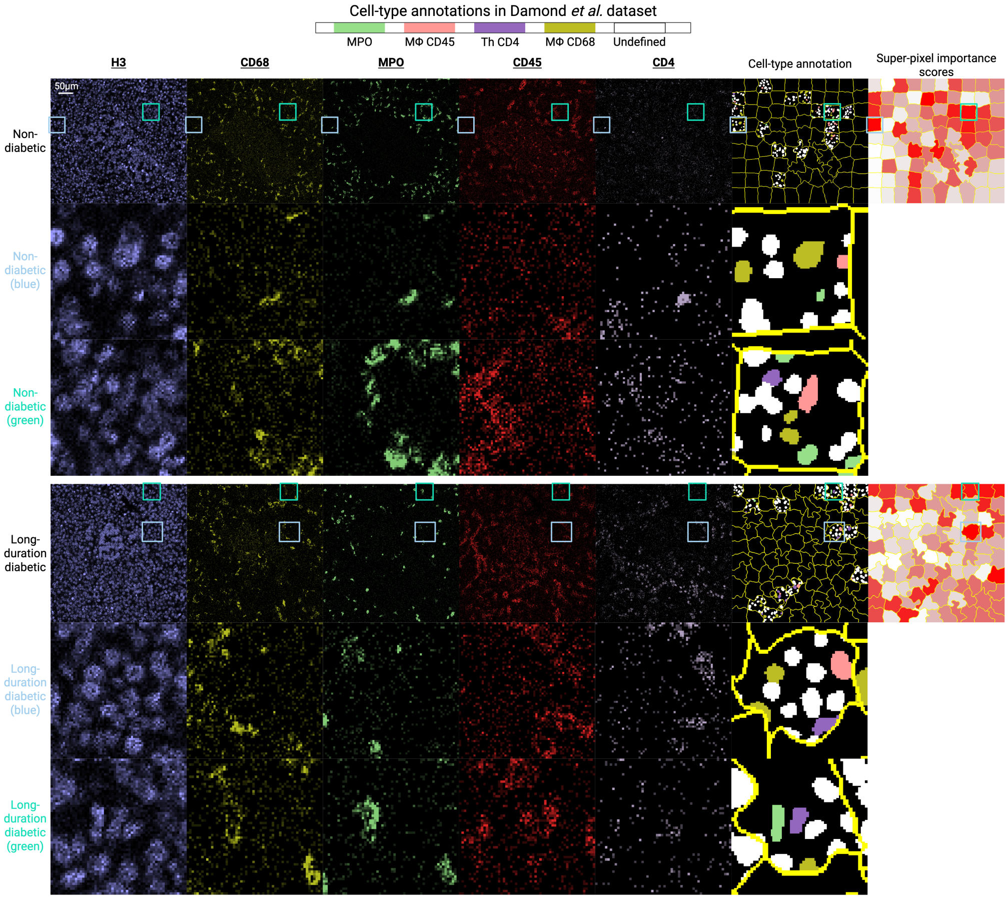

Fig. S4. Visualization of high-importance superpixel regions and associated immune cell markers in the Damond et al. diabetes dataset. Examples of superpixel regions with high importance scores predicted by `MICRON` are shown for non-diabetic and long-duration diabetic samples. Images show expression for individual markers, including H3, CD68, MPO, CD45, and CD4. H3 staining was used to identify cell nuclei. Immune cell populations were annotated based on canonical markers, including $CD68^{+}$ macrophages, MPO^{+} neutrophils, $CD45^{+}$ leukocytes, and $CD4^{+}$ T helper cells. Light-green and light-blue boxes mark two representative superpixels with high model importance that were prioritized as being outcome-associated by the `MICRON` framework. The cell-type annotation maps display the spatial distribution of immune cells in these regions. The samples were not arcsinh-transformed during preprocessing, which causes the cropped regions to appear more irregular in shape. Enlarged views highlight the presence and local organization of immune cell populations within the influential superpixel areas identified by `MICRON`.

Table S1. Notation used in `MICRON`.

Symbol	Description
D	The dataset containing all samples.
i	Index of a sample, where $i = 1, \ldots, I$.
I	Total number of samples in the dataset.
$\mathcal{B}_i$	Bag (collection) of instances for sample i.
r_i^m	The m-th instance in sample i.
m	Index of an instance within sample i, where $m = 1, \ldots, M$.
M	Total number of instances in sample i.
z_i	Class label for sample i.
z_i^m	Class label associated with the m-th instance in sample i, where $z_i^m = z_i$ for all m.
$h \times h \times N$	Dimensions of an input image, where $h \times h$ denotes spatial size and N is the number of channels.
$P_i^m \in \mathbb{R}^{h'_m \times h'_m \times K}$	Instance-level class probability tensor produced by the fully convolutional network for instance m in sample i.
h'_m	Spatial dimension of the class prediction tensor for instance m.
K	Total number of outcome classes. In this study, we consider binary classification (K = 2).
$P_{i,k}$	Collection of predictions for class k across all instances in sample i, defined as $P_{i,k} = \{P_{i,k}^1, \ldots, P_{i,k}^M\}$.
$P_{i,k}^m$	Prediction score for class k at instance m in sample i.
$\tilde{\mathcal{P}}_{i,k}$	Filtered and sorted vector of foreground prediction scores for class k in sample i.
$\lvert\tilde{\mathcal{P}}_{i,k}\rvert$	Cardinality in the filtered prediction vector $\tilde{\mathcal{P}}_{i,k}$.
Q	Number of quantiles used to summarize the distribution of instance-level predictions.
q	Quantile index, where $q = 1, \ldots, Q$.
$V_{i,k}$	Quantile vector for class k in sample i, which obtained from $\tilde{\mathcal{P}}_{i,k}$.
$v_{i,k}^{(q)}$	The q-th quantile value in the quantile vector $V_{i,k}$.
$\mathbf{V}_i$	Joint quantile feature vector for sample i, formed by concatenating quantile vectors from all classes and denoted as $\mathbf{V}_i = [V_{i,1}, \ldots, V_{i,K}]$.
S_i	Sample-level predicted class probability vector after applying softmax to $\mathbf{V}_i$.
$S_{i,k}$	Predicted probability that sample i belongs to class k.
$y_{i,k}$	Binary indicator encoding whether sample i belongs to class k.
$\mathcal{L}_{\mathrm{CE}}$	Categorical cross-entropy loss over all samples.
d	Dimension of the embedding vector for each crop.
$\mathbf{X}_{i,c} \in \mathbb{R}^{1 \times d}$	d-dimensional embedding vector extracted from the c-th crop in sample i.
$\boldsymbol{\phi}^{(i,c)}$	SHAP value vector for the embedding of crop c in sample i.
$\phi_j^{(i,c)}$	SHAP value of the j-th embedding dimension for crop c in sample i.
j	Index of an embedding dimension, where $j = 1, \ldots, d$.
$\mathrm{Importance}_{i,c}$	Importance score of crop c in sample i, computed as the sum of absolute SHAP values across all embedding dimensions.
